# Improving Catalytic Efficiency of L-Arabinose Isomerase from *Lactobacillus plantarum* CY6 towards D-Galactose by Molecular Modification

**DOI:** 10.3390/foods13111727

**Published:** 2024-05-31

**Authors:** Chengyu Lu, Ziwei Chen, Yuvaraj Ravikumar, Guoyan Zhang, Xinrui Tang, Yufei Zhang, Mei Zhao, Wenjing Sun, Xianghui Qi

**Affiliations:** 1 School of Food and Biological Engineering, Jiangsu University, 301 Xuefu Road, Zhenjiang 212013, China; 2 School of Life Sciences, Guangzhou University, 230 Wai Huan Xi Road, Guangzhou 510006, China

**Keywords:** D-tagatose, L-arabinose isomerase, site-directed mutagenesis, biotransformation

## Abstract

L-Arabinose isomerase (L-AI) has been commonly used as an efficient biocatalyst to produce D-tagatose via the isomerization of D-galactose. However, it remains a significant challenge to efficiently synthesize D-tagatose using the native (wild type) L-AI at an industrial scale. Hence, it is extremely urgent to redesign L-AI to improve its catalytic efficiency towards D-galactose, and herein a structure-based molecular modification of *Lactobacillus plantarum* CY6 L-AI (LpAI) was performed. Among the engineered LpAI, both F118M and F279I mutants showed an increased D-galactose isomerization activity. Particularly, the specific activity of double mutant F118M/F279I towards D-galactose was increased by 210.1% compared to that of the wild type LpAI (WT). Besides the catalytic activity, the substrate preference of F118M/F279I was also largely changed from L-arabinose to D-galactose. In the enzymatic production of D-tagatose, the yield and conversion ratio of F118M/F279I were increased by 81.2% and 79.6%, respectively, compared to that of WT. Furthermore, the D-tagatose production of whole cells expressing F118M/F279I displayed about 2-fold higher than that of WT cell. These results revealed that the designed site-directed mutagenesis is useful for improving the catalytic efficiency of LpAI towards D-galactose.

## 1. Introduction

D-tagatose is a rare monosaccharide which sparsely appears in the natural environment. The maximum applications of D-tagatose are found in the food and pharmaceutical industries because of its low caloric value (1.5 kcal/g) and 92% sweetness relative to sucrose [1,2]. Recently, studies investigating the therapeutic efficacy of D-tagatose have clearly mounted its hypoglycemic effect and ability to hinder the biofilm and plaque formation in dental caries [3,4]. Furthermore, the safety of its use in animals and humans and its beneficial gastrointestinal effects have also elucidated the possibility of D-tagatose use in pharmaceutical industries [5]. In virtue of its unique properties and proven safety for human health, the U.S. Food and Drug Administration (USFDA) in 2001 gave this rare sugar Generally Recognized as Safe (GRAS) status [4]. D-tagatose is also commonly used in cosmetics and detergents and is a primary precursor for synthesizing various optically active compounds [1,5]. The industrial importance of D-tagatose, combined with its potential benefits, has provoked the demand for D-tagatose production in the last decade. Due to its scarcity and low availability in natural sources [6], D-tagatose at the industrial level is usually produced by chemical method, patented by Biospherics Incorporated (USA), using D-galactose as a starting material [7]. This method consists of two steps: firstly, the D-galactose in the presence of sodium or calcium hydroxide is isomerized to form D-tagatose; secondly, the acid treatment is carried out, and D-tagatose is formed as an insoluble salt, which is further separated and purified. The cost involved in chemical synthesis remains a considerable burden, and industries are slowly shifting towards greener methods of producing D-tagatose via microorganisms or using microbial enzymes as biocatalysts.

Lately, the enzymatic platform for producing rare sugars using isomerases has earned attention among researchers as it will be an eco-friendly and inexpensive option in industrial settings [8]. In particular, L-arabinose isomerase (L-AI), due to its broad substrate specificity, can catalyze the isomerization of D-galactose to produce D-tagatose [9]. To date, L-AIs from various bacterial sources have been identified to be capable of synthesizing D-tagatose, particularly the strains belonging to the bacillus genus, such as *Bacillus* sp. [10], *Geobacillus* sp. [11], *Lactobacillus* sp. [12], *Alicyclobacillus* sp. [13]. In addition to mesophiles, the L-AIs from thermophilic bacteria viz. *Geobacillus* sp. [14], *Acidothermus* [15], and *Thermotoga* sp. were also found to be a potential source for D-tagatose biosynthesis [16]. Although there are plenty of L-AIs available, their activity towards D-galactose is often not significantly effective to be used at an industrial scale. This is mainly due to the L-AI’s substrate promiscuity nature and broad active site region, which allows it to accommodate non-canonical substrates like D-galactose and eventually lead to a reduction in catalytic activity [6]. Hence, the molecular modification of L-AI is mandatory to improve its performance. Additionally, the solved crystal structure of L-AI from mesophile and thermophilic microbes has deepened our understanding of the structure-functional activity while converting D-galactose to D-tagatose. For example, structural insights reveal that the L-AI from *Lactobacillus fermentum* exists as a hexamer (dimer of timers), and Mn^2+^ binding results in improved catalytic activity [17]. Being homodimer, the L-AI’s catalytic region is composed of a narrow space (tunnel-like interface), where the hydroxyl groups from Glu306 and Glu333, with the help of metal-mediated proton exchange, participate in converting D-galactose to D-tagatose via an enediol intermediate [11]. Inspired by the available seminal structural data, protein engineers constantly create novel engineered L-AI variants via rational design approaches to improve catalytic efficiency, stability against higher temperatures and broader pH, etc. In earlier reports, site-directed mutagenesis towards the active site area (H18T) in *Geobacillus stearothermophilus* resulted in 2.3-fold enhanced catalytic activity while isomerizing D-galactose [18]. Similarly, engineering the hot spot residues away from the active site of *L. fermentum* L-AI was also investigated. The results showed that mutating D268, D269, and D299 into K268, K269, and K299 enabled the mutant to exhibit good activity at acidic pH conditions (pH 5.0) [19]. The application of protein engineering tools remains a crucial factor in boosting the performance of L-AI towards non-canonical substrate D-galactose.

Although a few studies have been reported to improve the catalytic efficiency of L-AI towards D-galactose by molecular modifications, it still cannot remotely meet the needs of industrial production of D-tagatose. In this study, the enzymatic properties of L-AI (LpAI) from the food-grade lactic acid bacterium *Lactobacillus plantarum* CY6 was biochemically characterized. Additionally, the site-directed mutagenesis at defined sites lining the substrate binding pocket of LpAI was rationally designed to further improve the catalytic efficiency towards D-galactose, which is conducive to promoting the industrial production of D-tagatose.

## 2. Materials and Methods

### 2.1. Strains, Materials, and Plasmid Construction

The plasmid pQE-80L was purchased from Fenghui Biotechnology Co., Ltd. (Changsha, China), and *Escherichia coli* BW25113 was commercially obtained from HonorGene (Changsha, China). The strain *Lactobacillus plantarum* CY6 was derived from our previous work [20]. The DNA cloning, extraction, and purification kits were acquired from Vazyme Biotech Co., Ltd. (Nanjing, China). PCR primers (see Supplementary Material) were designed using SnapGene Viewer (v2.8.3, GSL Biotech, Chicago, IL, USA) and synthesized by GENEWIZ Biotechnology (Nanjing, China). The restriction endonucleases *SacI* and *SalI* for double digestion of plasmid pQE-80L were purchased from Takara Biomedical Technology Co., Ltd. (Beijing, China). The amplified *araA* gene was cloned into the linearized pQE-80L vector, named pQE80L-LpAI, and utilized for the expression of protein LpAI. The D-galactose, D-tagatose, L-ribulose standards, and isopropyl-β-D-thiogalactopyranoside (IPTG) were provided by Shanghai Aladdin Biochemical Technology, and the Ni^2+^-NTA resin was supplied by Sangon Biotech Co., Ltd. (Nanjing, China). Other chemicals were procured from Sinopharm Chemical Reagents (Shanghai, China).

### 2.2. Structural Modeling and In Silico Mutagenesis of LpAI

Based on the nucleotide sequence, the corresponding amino acid sequence was generated, and a BlastP search was carried out to obtain the different structures of L-AI. The multiple sequence alignment results reveal that *L. fermentum* CGMCC2921 shares maximum similarity with LpAI, and hence, the crystal structure of *L. fermentum* (PDB ID: 4LQL) was selected as the best template and employed for 3D modeling using SWISS-MODEL online server (https://swissmodel.expasy.org/, accessed on 8 July 2023). The output model structure was then verified using the Ramachandran plot generated by the PROCHECK (v.3.5.4, EBI, Cambridge, UK). By utilizing the modeled structure, we analyzed the substrate-lining binding pocket that exists in the interface region between the homodimers. In silico mutations were performed using Ser, Asn, Ile, Ala, Ser, Gly, Val, and Met as the targeted amino acids to replace the amino acids N278 (N278S), F279 (F279N, F279I), V365 (V365A), D369 (D369S, D369G), I370 (I370A, I370V), P420 (P420A, P420G), and F118 (F118M) via the mutagenesis tool present in PyMol.

### 2.3. Site-Directed Mutagenesis

The pQE-80L plasmid containing the LpAI gene was employed for generating mutants, e.g., LpAI_N278S_, LpAI_F279N_, LpAI_F279I_, LpAI_V365A_, LpAI_D369S_, LpAI_I370A_, LpAI_P420A_, LpAI_P420G_, LpAI_F118M_, and LpAI_F118M/F279I_. The LpAI mutants were generated using a whole plasmid polymerase chain reaction (PCR). The PCR protocol was as follows: pre-degeneration at 98 °C for 30 s, 32 cycles of degeneration at 98 °C for 10 s, annealing at 50 °C for 5 s, extension at 72 °C for 10 s, and finally, holding at 4 °C. Details of mutagenesis libraries and primer sequences are shown in Supplementary Table S1. The purified PCR products were assembled using a seamless linking kit (Vazyme, China). Subsequently, they were transformed into *E. coli* DH5α via the chemical transformation method and plated onto LB plates containing 100 mM ampicillin for overnight incubation at 37 °C. Sequencing of all mutants was outsourced to GENEWIZ Biotechnology (Suzhou, China).

### 2.4. Protein Expression and Purification

*E. coli* BW25113 transformants carrying pQE80L-LpAI and its mutants were grown aerobically at 37 °C and 220 rpm in 200 mL of LB medium inoculated with 100 mM ampicillin. Once the absorbance reached 0.6 at OD_600_, the protein expression was induced by adding 0.1 mM IPTG to the culture. The culture was then prepared for expression at 20 °C and 120 rpm overnight. After the induction period, the cells were collected by centrifugation (intelligent high-speed refrigerated centrifuge 3H16RI, Hunan Herexi Instrument & Equipment Co., Ltd., Changsha, China) at 8000 rpm and 4 °C for 4 min. Then rinsed twice with 10 mM phosphate-buffered saline (PBS, pH 7.4) and subsequently centrifuged again at 8000 rpm and 4 °C. The cell pellets were further resolubilized in 10 mM PBS and subjected to cell lysis using a sonicator (Nanjing Immanuel Instrument Equipment Co., Ltd., Nanjing, China) for 20 min with 3 s pulse and 5 s off settings. After sonication, the lysed cells were centrifuged at 10,000 rpm for 10 min at 4 °C. The soluble fractions were subsequently filtered using a 0.22 µM filter membrane, and the target enzyme was further purified using Ni^2+^-NTA affinity column chromatography at 4 °C following the manufacturer’s instructions. The purified enzyme fractions were collected and dialyzed against 1 × PBS buffer for 12 h to eliminate the residual imidazole that exist during the purification buffers. Finally, the sample was concentrated using 10 KD ultrafiltration tubing (Millipore, Nantong, China). The fractions that were eluted while purification was then analyzed by SDS-PAGE, and the purified enzymes (both WT and mutants) concentrations were determined using the BCA Protein Quantification Kit (Vazyme, China).

### 2.5. Determination of Enzyme Activity and Kinetic Parameter

To determine the LpAI activity of WT and mutants, the enzyme reaction (1 mL) was performed using 0.3 mg/mL of purified enzymes, 1 M of L-arabinose, 1 M of D-galactose, and 5 mM of MnSO_4_ in 10 mM PBS (pH 7.4) at 50 °C for 2 h. After 2 h, the samples were immediately placed in a boiling hot water bath for 10 min to stop the reaction. The L-ribulose and D-tagatose production was determined using HPLC chromatography equipped with a Hi-Plex Ca Column (300 × 7.7 mm, Agilent, Agilent Technologies, Santa Clara, CA, USA) and a refractive index detector (RID-20A) (Shimadzu, Tokyo, Japan). The mobile phase was deionized water at 84 °C with a 0.6 mL/min flow rate. The kinetic parameters such as *K_m_* (mM), *K_cat_* (min^−1^), and *V_max_* (U mg^−1^) for L-arabinose and D-galactose were identified using Michaelis–Menten plots and plotted with GraphPad Prism software (v8.0.1, GraphPad Prism Software, Inc., San Diego, CA, USA). All experiments were done in duplicates, and the results were expressed as mean ± standard deviation (SD).

### 2.6. Effect of Temperature, pH, and Divalent Metal Ions

To investigate the effect of temperature on LpAI WT and its mutants, an enzyme reaction containing 0.7 M of D-galactose, 5 mM of MnSO_4_, along with 0.1 mg/mL of purified enzyme was incubated in 10 mM PBS buffer (pH 7.4) at various temperatures (40 to 65 °C) for 5 h before measuring the activity. The pH effect was analyzed using three different buffer systems: 10 mM sodium acetate buffer (pH 4.0–6.0), 10 mM phosphate buffer (pH 6.0–8.0), and 10 mM glycine-sodium hydroxide buffer (pH 8.0–10.0) using the 0.7 M D-galactose, 5 mM of Mn^2+^, and 0.1 mg/mL purified enzymes at optimum temperature for 5 h. Finally, the role of different divalent metal ions in influencing the enzyme activity was assessed by incubating the enzyme assay containing 10 mM of Ca^2+^, Co^2+^, Mn^2+^, Cu^2+^, Fe^2+^, Zn^2+^, Ni^2+^, and EDTA, respectively along with the same concentrations of D-galactose, Mn^2+^ and purified enzymes at optimum pH and temperature for 5 h. Relative activities of the WT and its mutants for the effect of temperature, pH, and divalent metal ions are determined based on the amount of D-tagatose produced. All experiments were done in duplicates and are represented in graphs with standard deviations.

### 2.7. D-Tagatose Biosynthesis of Whole Cell

Initial screening using whole cell and the cysteine-carbazole sulfuric acid method was employed to identify the best D-tagatose-producing mutants. The recombinant *E. coli* BW25113 strains containing WT and mutants were cultured and expressed under optimum conditions as described in 2.4. The cells were then collected and washed twice with 10 mM PBS (pH 7.4) and resuspended again in 1 mL of PBS containing 150 g/L D-galactose and 5 mM of Mn^2+^ and allowed the reaction at 50 °C for 1 h. Followingly, the reaction was terminated by subjecting them to boiling water (100 °C) for 10 min, and the D-tagatose concentrations from each sample were measured at OD_560_. Further, the WT and double mutant LpAI_F118M/F279I_ expressed cells were used to demonstrate the potential application of whole cell as a biocatalyst for D-tagatose biosynthesis. Wet weight of about 100 g/L whole cells was taken in a reaction tube containing 250 g/L D-galactose, and 5 mM Mn^2+^ resuspended in 10 mM of sodium acetate buffer (pH 5.0). At each defined time interval, 1 mL of aliquot from the reaction sample was taken out, and the reaction was terminated by placing it in a boiling water bath for 10 min. The samples were then centrifuged, and D-tagatose concentrations were analyzed using HPLC with the conditions mentioned above. Triplicates were performed, and the results are expressed with standard deviations.

### 2.8. Molecular Docking Analysis

The modeled 3D structure of LpAI was used for molecular docking analysis. Initially, to identify the active site region in our modeled protein, the structure was superimposed with ligand co-crystallized L-AI from *Geobacillus kaustophilus* (PDB: 4R1Q), *L. fermentum* (PDB: 4LQL), and *E. coli* (PDB: 2AJT), which revealed the exact or speculated active site region and the functional residues. Three-dimensional structures of D-galactose were retrieved from the PubChem database (PubChem ID: 6036). Energy minimization and hydrogen atoms were added to the ligands using Discovery Studio 2019. Likewise, Gasteiger charges and non-polar hydrogens were added and merged into the modeled LpAI. Then docking was initiated using AutoDock Vina (v1.5.6, Avada, New York, NY, USA), and grid size was set up to 10 Å in the active site centered at x = −49.58, y = 34.637, z = −24.512 coordinates. The docking was performed, and the best-docked conformation was chosen based on the interactions formed by the E306, E333, H350, and H450 with D-galactose. The docked complexes were further exported in PDB format and are visualized for further analysis using PyMol (v2.5.2, Schrodinger Inc., New York, NY, USA) and Maestro (v2.0.0, Schrodinger Inc., New York, NY, USA).

### 2.9. Statistical Analysis

Significant variations among the experimental factors were determined by one-way ANOVA with Tukey post-hoc test, considering the significance level at *p* < 0.05. Comparison between two factors (e.g., two mutants) was performed by Student’s *t*-test (at *p* < 0.05). All analyses were done in GraphPad Prism software.

## 3. Results

### 3.1. Expression and Purification of LpAI

**Figure 1:**
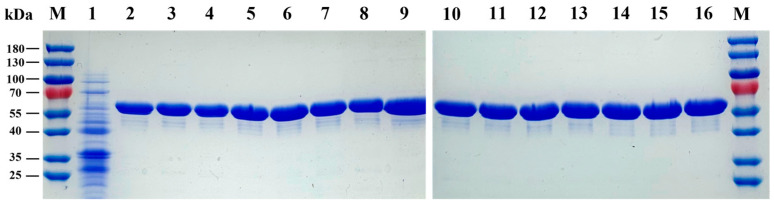
SDS-PAGE analysis of LpAI WT and mutants. Lane M, protein marker; lane 1, pQE-80L without LpAI; lane 2, WT; lane 3–16, N278S, F279N, F279I, V365A, D369S, D369G, I370A, I370V, P420A, P420V, F118M, F118M/F279I, F279I/P420G and F279I/F118M/P420G, respectively.

The plasmid carrying the LpAI encoding-gene (*araA*) was cloned into the pQE-80L vector and were transformed into *E. coli* BW25113. The recombinant cell harboring under IPTG induction led to overexpression of LpAI. The concentrated LpAI was then analyzed using SDS-PAGE. The SDS-PAGE analysis revealed that the LpAI was well overexpressed with molecular weight of 55.5 kDa (Figure 1).

### 3.2. Biochemical Characterization of LpAI

#### 3.2.1. Effect of Metal Ion

**Figure 2:**
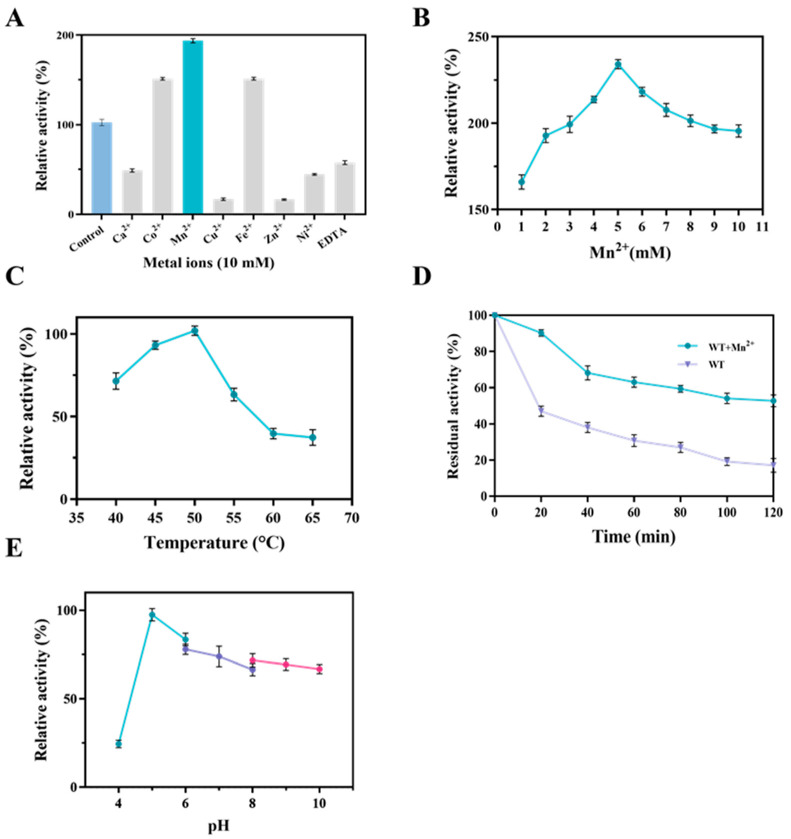
Effects of metal ions, temperatures, and pH on the activity of LpAI. (**A**) Effect of metal ions on enzymatic activity. The activity of the control without any added metal ions was defined as 100%. (**B**) Effect of Mn^2+^ concentration on enzyme activity. Activity without the addition of Mn^2+^ was defined as 100%. (**C**) Effect of temperature on enzymatic activity. (**D**) Thermal stability of LpAI at 50 °C. (**E**) Effect of pH on enzymatic activity.

Various bacterial L-AIs reported to date are metal ion dependent, particularly manganese ion, which is found to play a vital role in boosting D-galactose isomerization [6]. LpAI activity was explored by conducting the enzyme assay using different divalent metal ions. The results demonstrated that the addition of 5 mM of Co^2+^, Mn^2+^, and Fe^2+^ can significantly increase the relative activity of LpAI (Figure 2A). Adding Co^2+^ and Fe^2+^ exerted a 51% improvement in enzyme activity, whereas maximum activity (94%) was found with the Mn^2+^ added samples. Other metal ions such as Co^2+^, Cu^2+^, Zn^2+^, and Ni^2+^ significantly hampered the enzyme activity. Adding Cu^2+^ and Zn^2+^ drastically diminished the enzyme activity, where almost substantial activity levels could not be measured. With manganese’s significant and intrinsic ability to improve LpAI activity, additional studies were performed using different concentrations of Mn^2+^.

As shown in Figure 2B, it is evident that increasing Mn^2+^ concentration results in increasing enzyme activity, and the optimum Mn^2+^ concentration was found to be 5 mM, where the highest enzyme activity (234%) was measured. However, a further rise in Mn^2+^ concentration led to a reduction in activity. However, it was not so significant as only 38% of activity was reduced at 10 mM Mn^2+^. Together, these results indicate that the LpAI activity toward isomerizing D-galactose is firmly Mn^2+^ dependent, which is consistent with the results of the earlier findings.

#### 3.2.2. Effect of Temperature and pH

The effect of temperature on the catalytic activity of LpAI was determined based on a thermostability assay. The WT activity in the presence of Mn^2+^ was assessed over a range of 40 to 65 °C, where the maximum activity was found at 50 °C. As shown in the temperature profile (Figure 2C), the WT showed increased activity until 50 °C; however, as the temperature rose further, the enzyme activity was significantly reduced. Around 50% activity was reduced at 55 °C, and 64% at 65 °C. The thermal stability of LpAI at 50 °C in the presence of 5 mM Mn^2+^ was also measured. It revealed that a sharp decline in enzyme activity was noted in the absence of Mn^2+^. Almost 50% of activity was lost in the first 20 min (Figure 2D), and the residual activity decreased by 80.9% at 100 min. On the contrary, in the presence of 5 mM Mn^2+^, the LpAI retained 90.3% activity for the first 20 min, and the enzyme stability was increased with a half-life of about 120 min at 50 °C. Furthermore, at 120 min, the residual activity of LpAI in presence of Mn^2+^ was 35.6% higher than that of LpAI in absence of Mn^2+^. Next, the effects of pH on LpAI activity were examined. The WT exhibited excellent activity in slightly acidic conditions (pH 5.0 and 6.0), where the highest activity was seen at pH 5.0, and 83.5% activity was retained at pH 6.0 (Figure 2E).

### 3.3. Rational Design to Develop a Highly D-Galactose Isomerizing LpAI

#### 3.3.1. Multiple Sequence Alignment, Homology Modeling, and Active Site Analysis

**Figure 3:**
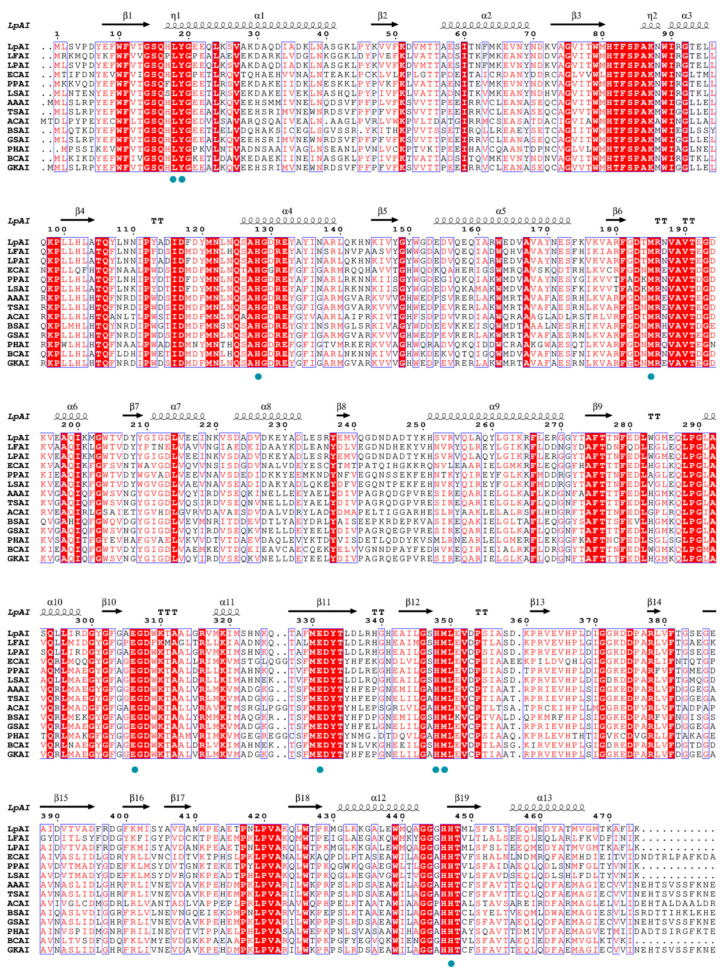
Multiple sequence alignments of L-AIs from various sources. LpAI (*Lactobacillus plantarum* CY6), LFAI (*Lactobacillus fermentum* CGMCC2921), LPAI (*Lactobacillus plantarum* NC8), ECAI (*Escherichia coli* W3100), PPAI (*Pediococcus pentosaceus* PC-5), LSAI (*Lactobacillus sakei* 23K), AAAI (*Alicyclobacillus acidocaldarius*), TSAI (*Thermus* sp. IM6501), ACAI (*Acidothermus cellulolytics* 11B), BSAI (*Bacillus subtilis*), GSAI (*Geobacillus stearothermophilus*), PHAI (*Pseudoalteromonas haloplanktis* ATCC14393), BCAI (*Bacillus coagulans* NL01), GKAI (*Geobacillus kaustophilus*). The black wavy lines and arrows represented α-helix and β-strand, respectively. In all the sequences, amino acid residues that are identical are highlighted in red and those that are highly conserved and similar are framed in blue. Catalyzed functional residues are marked with green dots.

The biochemical characterization shows that WT is not highly efficient towards D-galactose as it is a non-canonical substrate. Thus, to improve the D-galactose isomerization efficiency of LpAI, the rational-based site-directed mutagenesis was performed based on the structural information. As shown in Figure 3, the multiple sequence alignment (MSA) analysis for LpAI was carried out with 13 L-AIs from various sources (mesophiles and thermophiles).

**Figure 4:**
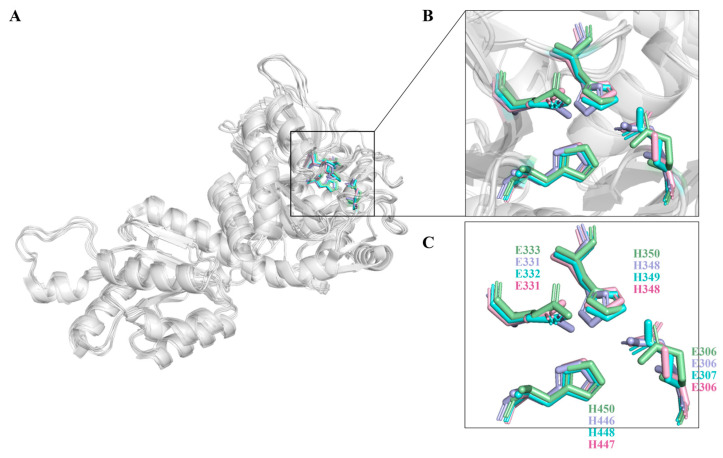
Structure aligns with 4R1Q, 4LQL, and 2AJT crystals. Pink in the figure is LpAI. (**A**) Stacked 3D structure of L-AIs monomers. (**B**,**C**) Superimposed active site regions of different L-AI structures. Purple (E306, E331, H348 and H446), aquamarine (E307, E332, H349 and H448) and green (E306, E333, H350 and H450) in the figure are 4LQL, 4R1Q and 2AJT, respectively.

The amino acid sequence identity between LpAI and other characterized L-AIs is shown in Table S2. The MSA analysis found 70.5% and 70.7% sequence similarity in *Lactobacillus* sp. and *Pediococcus* sp. It showed that the active residues H350, H450, E306, and E333 in LpAI are strictly conserved as in Figure 3. Additionally, to deduce any differences in amino acid near the active site or in the entrance of the substrate binding pocket, we modeled the LpAI with the similarly identical L-AI from *L. fermentum*. The *L. fermentum* crystal structure (PDB ID: 4LQL) was chosen as a template, and homology models were performed. The modeled LpAI structure was superimposed with the crystal structures of *G. kaustophilus*, *L. fermentum* and *E. coli* L-AIs (Figure 4). The spatial orientation of active residues E306, E333, H450 and H350 in LpAI was checked. The other residues that help in substrate positioning and stabilization are L18, Y19, M185, F279, and I371.

#### 3.3.2. Molecular Modification of LpAI

**Figure 5:**
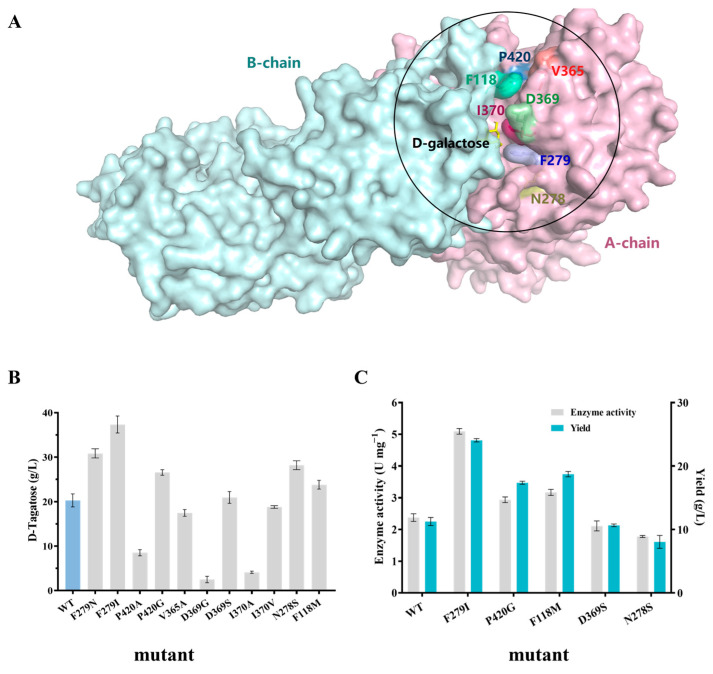
Yield and enzyme activity of mutants. (**A**) Substrate channels of LpAI after docking with D-galactose. (**B**) Primary screening of single point mutants by whole cell method. (**C**) Validation of enzyme activity in single point mutants. The error bars are described the as average ± standard deviation (SD).

With the structural insights from earlier reports, we selected the amino acids surrounding the 8 Å residues from the active site and the residues that help in substrate-guiding to the active site. Accordingly, F118, N278, F279, V365, D369, I370, and P420 were chosen and subjected to site-directed mutagenesis (Figure 5A). To engineer the aforementioned amino acids and to facilitate the D-galactose binding with higher specificity, the following mutants were generated: N278S, F279N, F279I, V365A, D369S, D369G, I370A, I370V, P420A, P420G, and F118M, respectively. Whole cell expressing LpAI mutant was screened for D-tagatose production. The cysteine carbazole-based analysis method revealed that five out of eleven mutants exhibited higher D-tagatose production than WT (Figure 5B). The F279I mutant showed the highest D-tagatose yield (37.35 g/L), followed by F279N (30.85 g/L), N278S (28.20 g/L) and P420G (26.56 g/L). Albeit insignificant (*p* > 0.05), a slight increase in D-tagatose yield was observed in F118M mutant (23.80 g/L). On the other hand, when compared to the yield of WT (20.27 g/L), mutating I370 into Val, V365 to Ala, and P420 to Ala reduced D-tagatose production. The D369G (2.49 g/L) and I370A (4.12 g/L) mutants largely led to a maximum loss in D-tagatose synthesis, as shown in Figure 5B. Mutating Asp at 369 into Ser has not significantly changed the D-tagatose yield (*p* > 0.05). Further, the mutant enzymes were purified to demonstrate the best mutants, and the specific activity and D-tagatose yield were calculated. The enzyme activity was measured in 10 mM PBS (pH 7.4) containing 125 g/L D-galactose and 5 mM Mn^2+^ at 50 °C for 5 h. As depicted in Figure 5C, the enzyme activity of F279I, P420G, and F118M was elevated by 111.0% (5.00 U mg^−1^), 24.1% (2.94 U mg^−1^), and 37.1% (3.25 U mg^−1^), respectively, compared to WT (2.37 U mg^−1^).

#### 3.3.3. Iterative Mutation Characterization and Kinetic Parameters

**Figure 6:**
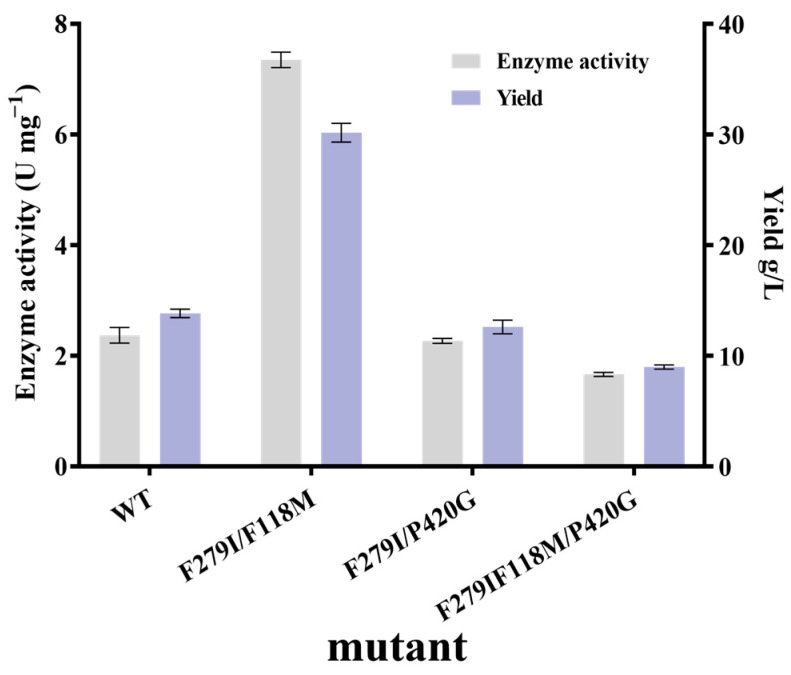
Yield and enzyme activity of combinatorial mutants. The error bars are described the as average ± standard deviation (SD).

As the mutants F279I, P420G, and F118M showed a boosted specific activity, combined mutations of these three residues were executed to further demonstrate the feasibility of improving the catalytic functions and the beneficial synergistic effects. Hence, double and triple mutants are developed, which are mutants F118M/F279I, F279I/P420G, and F279I/F118M/P420G (Figure 6). The double mutant F118M/F279I displayed a pronounced specific activity (7.35 U mg^−1^ vs. 2.37 U mg^−1^) and higher D-tagatose yield (30.17 g/L vs. 13.83 g/L) when compared to WT. On the other hand, F279I/P420G and F279I/F118M/P420G failed to exhibit any improvements in enzyme activity and D-tagatose conversion efficiency.

**Figure 7:**
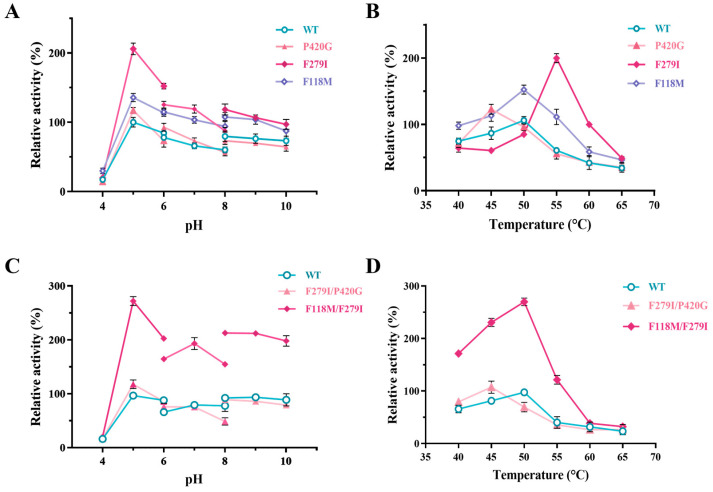
Effect of different pH and temperature on the mutant enzyme. (**A**,**B**) The trends of enzyme activities of single point mutants subjected to different pH and temperatures. (**C**,**D**) The trends of enzyme activities of the combined mutants subjected to different pH and temperatures. The optimal activity of WT was defined as 100% and the relative activity of the mutant enzymes under other conditions was calculated. The error bars are described the as average ± standard deviation (SD).

Subsequently, the effects of pH and temperature on the evolved enzymes were tested. It was shown that mutants F279I, P420G, F118M, F118M/F279I, and F279I/P420G had not exerted any changes in optimal pH, as the optimal pH for all these mutants were at pH 5.0. Nevertheless, increases in pH tolerance were noted, particularly in F118M, F279I, and F118M/F279I, where activities improved by 36% (U mg^−1^), 106% (U mg^−1^), and 172% (U mg^−1^) compared to WT (Figure 7A,C). The effects of temperature on mutates was analyzed under the condition of 40 to 65 °C. Among all the tested mutants, only F118M showed elevated activity across all temperatures (Figure 7B). The mutant P420G and F279I exhibited ~3-fold more activity at 45 °C and 55 °C, respectively, and F118M/F279I showed nearly ~2-fold enhanced activity at 50 °C. Overall, from both the temperature and pH profile, the double mutant F118M/F279I is comparatively much higher and can be a viable option to be used for D-tagatose synthesis applications.

**Figure 8:**
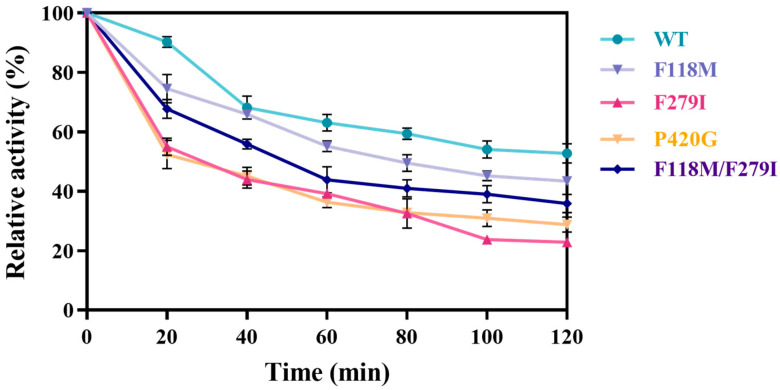
Thermal stability of WT and mutant enzymes. The activity of each enzyme that was not placed for a 2 h reaction at 50 °C was defined as 100%, and the relative enzyme activity was calculated for different placement times. The error bars are described the as average ± standard deviation (SD).

Additionally, to obtain insights into thermal stability, the mutants were assessed by performing an enzyme assay at 50 °C for 120 min. All mutants showed different degrees of diminished thermal stability, with P420G and F279I displaying the most significant loss (Figure 8). F118M/F279I exhibited reduced thermodynamically stability, possibly due to multiple phenylalanine substitutions. It has been noted that anion–aromatic interactions are more stable than other residue–residue interactions in protein structures [21].

To determine any improvements in catalytic activity of the generated mutants, the kinetic parameters such as *V_max_* and *K_cat_*/*K_m_* were evaluated (Table 1). From the results, it is evident that the *V_max_* and *K_ca_*_t_/*K_m_* values of the F118M/F279I mutant were 20.88 U mg^−1^ and 0.53 min^−1^ mM^−1^, respectively, which were 2.87-fold and 3.12-fold higher compared with the WT (7.27 U mg^−1^ and 0.17 min^−1^ mM^−1^). Notably, a remarkable reduction in the *K_m_* values were observed in all four mutants, particularly with the F118M/F279I mutant, where a reduction of up to 300 mM when compared to WT. The kinetic parameters in the double mutant showed higher activity with D-galactose than L-arabinose.

Enzyme activity of two different substrates was determined and compared to the WT. As shown in Table 2, the specific activities of all mutants towards L-arabinose have no significant change compared to WT. However, the specific activity of double mutant F118M/F279I towards D-galactose was increased by 210.1% compared to WT, which is obviously higher than its specific activity towards L-arabinose. Additionally, the F118M and F279I mutants also showed enhanced catalytic activity towards D-galactose compared to WT.

### 3.4. Molecular Docking and Whole Cell Biocatalytic Synthesis of D-Tagatose

**Figure 9:**
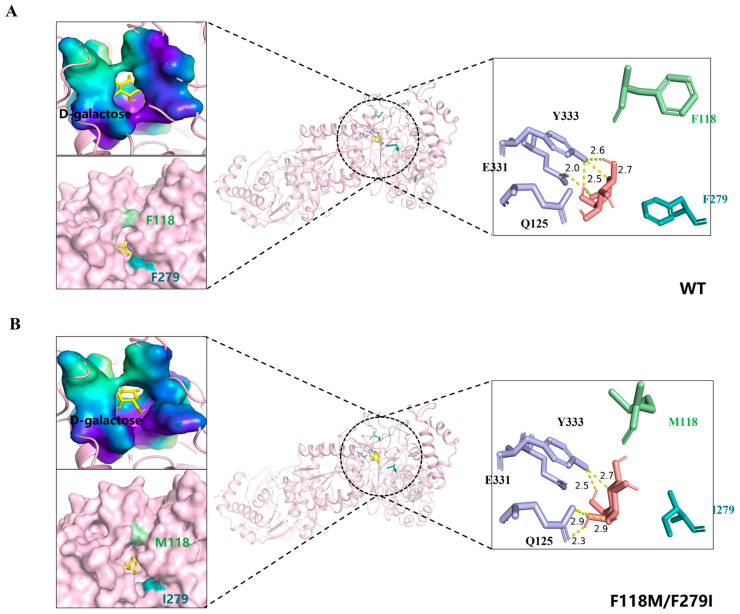
Residue distributions and binding pockets of WT and mutant enzyme. (**A**,**B**) Models of WT and F118M/F279I. D-galactose in yellow (left) and salmon color (right). The purple stick model indicates residues with interactions, and the yellow dashed line indicates hydrogen bonding interactions in angstroms (Å). The surface structures on the left side of the figure are the substrate-binding pocket (top) and substrate channel (bottom) of WT and F118M/F279I, respectively.

To elucidate the underlying principles governing the modified substrate preference and enhanced catalytic efficiency of mutant F118M/F279I, molecular docking was performed with generated mutant model F118M/F279I and WT (Figure 9). The active site analysis of the D-galactose-docked complexes in double mutants revealed that Q125 and Y333 form a hydrogen bond interaction with D-galactose. Unlike in WT, E331 with Y333 forms a hydrogen bond with D-galactose. The improved activity in the double mutant may be attributed to the residue Q125, which helps favor two H-bonds in binding with the substrate. Surface structure in Figure 9 (top left) shows the enlarged substrate-binding pocket of D-galactose, where the larger accommodation space and more hydrogen bonding might improve the specificity of F118M/F279I for D-galactose. Meanwhile, it was reasoned that substituting two smaller residues enlarged the substrate channel of LpAI (surface structure at lower left in Figure 9), alleviating the resistance to D-galactose transport.

**Figure 10:**
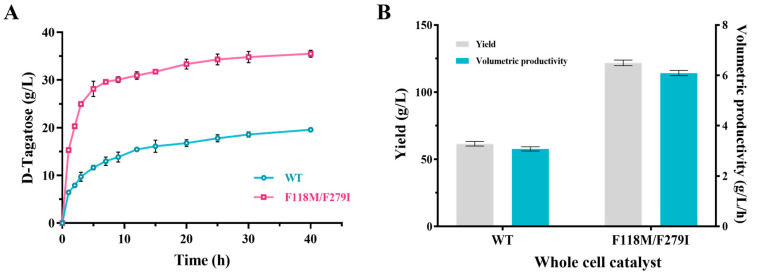
Comparison of D-tagatose production of WT and F118M/F279I. (**A**) D-tagatose production by pure enzyme. (**B**) Whole cell biotransformation for producing D-tagatose. The error bars are described the as average ± standard deviation (SD).

Given the mechanistic insights behind the improved catalytic performance, we attempted to demonstrate D-tagatose biosynthesis. As shown in Figure 10A, the conversion ratio (slope) of F118M/F279I was significantly increased in the first 5 h. After 8 h, the yield of the mutant continued to grow, while the WT reached a stabilization period. After 20 h, F118M/F279I and WT showed a similar increasing trend. The delayed increase in D-tagatose production after 20 h represented the optimal benefit time point for maximizing D-tagatose yield. When the reaction reached equilibrium, the D-tagatose yield and conversion ratio of WT were calculated to be 19.60 g/L and 15.7%, respectively. Compared to WT, the F118M/F279I mutant exhibited a higher D-tagatose yield (35.52 g/L) and conversion ratio (28.2%). To obtain a higher concentration of D-tagatose, we increased the concentration of the substrate D-galactose (250 g/L). Taken together, under optimized conditions of 50 °C and pH 5.0, recombinant *E. coli* BW25113 strains overexpressing F118M/F279I achieved higher D-tagatose production in 20 h compared to the use of F118M/F279I pure enzyme solution (Figure 10B). A total of 123.25 g/L of D-tagatose was obtained from 250 g/L of D-galactose, which is 1.96 times higher than that of WT (62.76 g/L). Moreover, the recombinant cell containing F118M/F279I exhibited a conversion ratio of 49.3% and a productivity of 6.2 g/L/h, which were significantly higher than those of WT (conversion ratio of 25.1% and productivity of 3.1 g/L/h).

## 4. Discussion

Usually, LpAI prefer to use L-arabinose as canonical substrate than D-galactose. To produce D-tagatose efficiently, LpAI with good catalytic activity for D-galactose is necessary on an industrial scale. Therefore, this study aims to enable the LpAI as a D-galactose preferred substrate through rational-based enzyme engineering involving the mutation of active site residues and amino acids lining the substrate binding. The molecular modification of LpAI yielded various mutants such as N278S, F279N, F279I, V365A, D369S, D369G, I370A, I370V, P420A, P420G, F118M, and double mutant F118M/F279I respectively. Interestingly, the results showed that substituting aromatic F279 residue with the hydrophobic residue Ile rendered better activity (111.0% higher activity). Similar results were documented in other sugar isomerases, and the replacement of hydrophobic residues in the active site with hydrophilic residues resulted in a change in substrate preference [22,23,24]. Except the F279I mutation, the F118M mutation showed improved activity of up to 37.1% (U mg^−1^). The higher activity mutants were combined to investigate any synergistic effects that help render much higher activity than single individual mutants. As expected, the derived double mutant activity increased 2.1-fold when compared to the WT. This clearly showed that these mutations have likely altered the substrate preference of LpAI, shifting it from L-arabinose to D-galactose. Similar activity changes or improvements in activities were also noted in other engineered L-AIs. For instance, in the L-AI of *B. coagulans* [22], *Shewanella* sp. [23], and *G. thermodenitrificans* strains [24], phenylalanine at 279 (280) has been attributed as a substrate preference switch for L-AI. Likewise, in 2018, a similar change in preference occurred in the H18T mutant from GSAI [18], which led to a higher ration of D-tagatose conversion in the presence of borate. In this study, the higher affinity of the F118M/F279I mutant towards D-galactose might be due to an additional H-bond formed with the catalytic residue Q125.

Generally, the bioconversion of D-galactose to D-tagatose by L-AI is favored at higher temperatures, unlike the bioconversion of L-arabinose to L-ribulose at mesophilic temperatures. Hence, it is important to investigate the stability of LpAI mutant at higher temperatures. Although the double mutant F118M/F279I produced higher enzyme activity, its thermostability at higher temperatures (50 °C) was obviously weakened. The reduced thermal stability observed in F118M/F279I may be attributed to the negative synergistic effect of multiple phenylalanine substitutions [24], leading to perturbations in the original protein structure and residue interactions. To establish the sustainable and stable production of D-tagatose by LpAI, alternative approaches may need to be considered in order to systematically address its thermal stability issue, such as enzyme immobilization and rational design strategies for protein flexible regions, etc. In addition, the P420G mutant showed a promising pH tolerance at pH 5.0, which is helpful for the isomerization of enzyme and the production of D-tagatose. The remaining mutants also exhibited enhanced pH tolerance. In particular, the higher enzyme activity exhibited by F118M/F279I in an acidic background may give it greater potential in the industrial production of D-tagatose.

Finally, the modified enzyme LpAI_F118M/F279I_ significantly improved its catalytic efficiency. F118M/F279I demonstrated superior D-tagatose synthesis by whole cell catalysis and achieved D-tagatose production at concentrations up to 123.25 g/L. Certainly, D-tagatose can be further separated from the system using sequential simulated moving bed chromatographic separation (SSMB) technology [25,26]. In addition, by utilizing Saccharomyces cerevisiae for selective degradation of residual substrates (e.g., D-galactose) [27,28], high purity D-tagatose can be obtained.

## 5. Conclusions

In summary, the combinatorial mutant F118M/F279I, one at the active site and the other at the substrate entry channel, showed increased catalytic activity and substrate specificity for D-galactose. The results demonstrated that structure-guided mutagenesis in the substrate lining region is an efficient strategy for developing enzymes that could catalyze the non-canonical substrate D-galactose. Furthermore, the whole cell of *E. coli* containing F118M/F279I mutant efficiently produced 123.25 g/L D-tagatose from 250 g/L D-galactose, with a higher conversion ratio of 49.3% than WT. Even so, further optimizations of fermentation conditions should be carried out to improve the production of D-tagatose. This study provides a good reference for the efficient biosynthesis of D-tagatose on an industrial scale.

## Figures and Tables

**Table 1:** Kinetics of WT and mutant enzymes.

Enzyme	*K_m_* (mM)	*V_max_* (U mg^−1^)	*K_cat_* (min^−1^)	*K_cat_*/*K_m_* (mM^−1^ min^−1^)
WT	1338.01	7.27	227.19	0.17
F118M	1229.88	9.03	334.33	0.27
F279I	1271.62	16.46	576.59	0.45
P420G	1305.30	8.31	230.75	0.18
F118M/F279I	1013.05	20.88	535.38	0.53

**Table 2:** Comparison of activities toward L-arabinose and D-galactose.

Enzyme	Specific Activity (U mg^−1^)		Ratio ^a^ (%)
	D-Galactose	L-Arabinose	
WT	2.37	3.79	62.53
F118M	3.25	3.53	92.07
F279I	5.00	3.35	149.25
P420G	2.94	3.73	78.82
F118M/F279I	7.35	3.46	212.43

^a^ The specific activity towards D-galactose/the specific activity towards L-arabinose.

## Data Availability

The original contributions presented in the study are included in the article/Supplementary Material, further inquiries can be directed to the corresponding authors.

## Supplementary Materials

The following supporting information can be downloaded at: https://www.mdpi.com/article/10.3390/foods13111727/s1, Table S1: The primers used in this study. Table S2: Comparison of amino acid sequence homology of the LpAI and reported L-AIs enzymes from various microorganisms.
