# Supplementary material for: Improving Catalytic Efficiency of L-Arabinose Isomerase from Lactobacillus plantarum CY6 towards D-Galactose by Molecular Modification
Source: Foods. 2024 May 31;13(11):1727. doi: 10.3390/foods13111727 (PMC11172353; doi:10.3390/foods13111727)
Supplement: Supplementary file 1 [file foods-13-01727-s001.zip › foods-3011748-supplementary.pdf]

Supplementary materials

Improving Catalytic Efficiency of L-Arabinose Isomerase from *Lactobacillus plantarum* CY6 towards D-Galactose by Molecular Modification

Chengyu Lu [1,†], Ziwei Chen [1,†], Yuvaraj Ravikumar [1], Guoyan Zhang [1], Xinrui Tang [1], Yufei Zhang [1], Mei Zhao [1], Wenjing Sun [1,*] and Xianghui Qi [1,2,*]

[1] School of Food and Biological Engineering, Jiangsu University, 301 Xuefu Road, Zhenjiang 212013, China

[2] School of Life Sciences, Guangzhou University, 230 Wai Huan Xi Road, Guangzhou 510006, China

* Correspondence: juswj@ujs.edu.cn (W.S.); qxh@ujs.edu.cn (X.Q.);
Tel./Fax: +86-511-88780201 (X.Q.)

[†] These authors contributed equally to this work.

Table S1. The primers used in this study

Name	Primer (5' – 3')
**Gene amplification**	
araA-F	GTATCCGCATGCGAGCTATGTTATCAGTACCTGATTATGAGT
araA-R	CTTGGCTGCAGGTCGATTACTTTAAGAATGCCTTAGTCAT
**Site-directed mutagenesis**	
N278S-F	TACCACGagcTTTGAAGATCTTTGGGGGATGG
N278S-R	CTTCAAAgctCGTGGTAAAGGCTGTGTAACCG
F279N-F	CACGAACaatGAAGATCTTTGGGGGATGGAGC
F279N-R	GATCTTCattGTTCGTGGTAAAGGCTGTGTAACC
F279I-F	CACGAACattGAAGATCTTTGGGGGATGGAGC
F279I-R	GATCTTCaatGTTCGTGGTAAAGGCTGTGTAACC
V365A-F	ATGGATGtgcTTCGACCCGTGGTTTATCACTT
V365A-R	GGTCGAAgcaCATCCATTGGATATTGGGGGTA
D369S-F	TCCATTGagcATTGGGGGTAGAGATGATCCTGC
D369S-R	CCCCAATgctCAATGGATGAACTTCGACCCGT
D369G-F	TCCATTGggtATTGGGGGTAGAGATGATCCTGC
D369G-R	CCCCAATaccCAATGGATGAACTTCGACCCGT
I370A-F	ATTGGATgcaGGGGGTAGAGATGATCCTGCTC
I370A-R	TACCCCCtgcATCCAATGGATGAACTTCGACC
I370V-F	ATTGGATgttGGGGGTAGAGATGATCCTGCTC
I370V-R	TACCCCCaacATCCAATGGATGAACTTCGACC
P420A-F	CCTAATTTAgcaGTTGCTAAGCAATTATGGACCCC
P420A-R	CTTAGCAACtgcTAAATTAGGTGTTTCGGCTTCTGG
P420G-F	CCTAATTTAggtGTTGCTAAGCAATTATGGACCCC
P420G-R	GCAACaccTAAATTAGGTGTTTCGGCTTCTGG
F118M-F	GCAGACATTGACatgGATTACATGAACCTTAACCAAAGTGC
F118M-R	TGTAATCcatGTCAATGTCTGCATATGGAATATTATTC
F118M/F279I-F	GCAGACATTGACatgGATTACATGAACCTTAACCAAAGTGC
F118M/F279I-R	GATCTTCaatGTTCGTGGTAAAGGCTGTGTAACC

Table S2. Comparison of amino acid sequence homology of the LpAI and rep orted L-AIs enzymes from various microorganisms

Enzyme	Genbank Accession no.	Identity (%)
LFAI	HM150718	67.3
LPAI	AL935262	99.8
ECAI	BAB96631	45.1
PPAI	AEM17146	70.7
LSAI	CAI56163	70.5
AAAI	AAY68209	53.6
TSAI	AY225311	53.8
ACAI	ACZ67491	45.4
BSAI	AOR99172	51.9
GSAI	ABY84698	54.2
PHAI	OLF74597	43.9
BCAI	KX356659	65.0
GKAI	BAD76189	54.00
LFAI	HM150718	67.3
LPAI	AL935262	99.8
ECAI	BAB96631	45.1
PPAI	AEM17146	70.7

Source of L-AIs: LFAI (*Lactobacillus fermentum* CGMCC2921), LPAI (*Lactobacillus plantarum* NC8), ECAI (*Escherichia coli* W3100), PPAI (*Pediococcus pentosaceus* PC-5), LSAI (*Lactobacillus sakei* 23K), AAAI (*Alicyclobacillus acidocaldarius*), TSAI (*Thermus sp.* IM6501), ACAI (*Acidothermus cellulolytics* 11B), BSAI (*Bacillus subtilis*), GSAI (*Geobacillus stearothermophilus*), PHAI (*Pseudoalteromonas haloplanktis* ATCC14393), BCAI (*Bacillus coagulans* NL01), GKAI (*Geobacillus kaustophilus*).
